# Delayed Diagnosis of Primary Hyperparathyroidism: A Case Report

**DOI:** 10.7759/cureus.49383

**Published:** 2023-11-25

**Authors:** Mónica Maria Silva

**Affiliations:** 1 Family Medicine, Unidade de Saúde Familiar (USF) Dr. Pelaez Carones, Braga, PRT

**Keywords:** osteoporosis, parathyroid gland adenoma, acute obstructive pyelonephritis, nephrocalcinosis, primary hyperparathyroidism

## Abstract

Primary hyperparathyroidism (PHPT) is characterized by an elevation in serum calcium levels, sometimes leading to aggravated clinical conditions, namely nephrolithiasis, nephrocalcinosis, and/or fractures. A 55-year-old patient was admitted to the hospital with acute obstructive pyelonephritis in March 2021, having another episode one year later. Initial blood and urine analysis detected inflammatory markers, namely C-reactive protein, and the presence of leucocytes and blood in the urine. The renal computed tomography scan exhibited renal asymmetry, nephrocalcinosis, and multiple kidney stones. The patient was scheduled for a follow-up one year later to perform blood and urine analysis to uncover the cause of nephrocalcinosis, displaying high serum calcium and parathyroid hormone (PTH) levels. The thyroid ultrasound revealed a parathyroid adenoma, which was removed through a right lower parathyroidectomy, improving the symptoms.

The clinical condition described here is an atypical manifestation of this disease because PHPT is normally asymptomatic. In the present case study, nephrocalcinosis and nephrolithiasis were strong indicators of the underlying disease. However, the delay in the follow-up consultation resulted in complications for the patient, such as microabscesses in the kidneys, which could lead to reduced renal function in the future. Early detection of key aspects of the disease could avoid further complications and suffering for the patient. For example, the family physician's follow-up of the patient’s condition could surpass the waiting time between consultations with different specialties, and promote early treatment.

## Introduction

Primary hyperparathyroidism (PHPT) is an endocrine disorder caused by excessive parathyroid hormone (PTH) production that significantly increases calcium levels in blood serum [1,2]. This disorder can be asymptomatic, mostly discovered with incidental multichannel screening [3,4]. However, some patients exhibit symptoms that can lead to an early diagnosis. Herein, we describe the case of a patient with a history of urinary tract infection (UTI) and two episodes of acute obstructive pyelonephritis that later progressed to urosepsis. After careful clinical examination and laboratory analysis, she was diagnosed with PHPT.

## Case presentation

**Figure 1:**
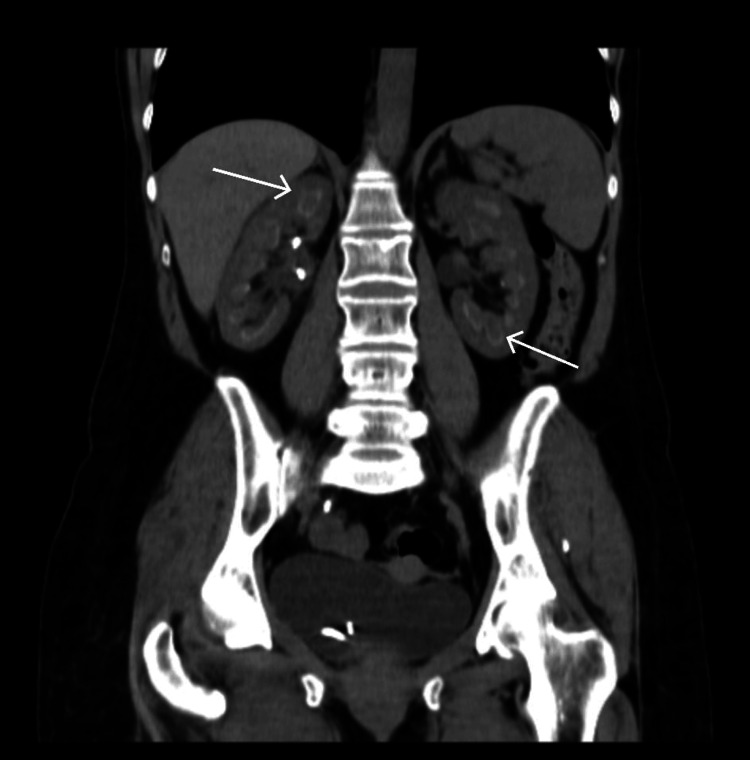
CT showing bilateral nephrocalcinosis (arrows)

A 55-year-old woman with a medical history of depression and dyslipidaemia was admitted to the hospital in March 2021 for right lumbar pain, dysuria, and polyuria. The patient was hypotensive, afebrile and had right costovertebral angle tenderness. Her creatinine and C-reactive proteins levels were high, at 1.9 mg/dL and 302.7 mg/L, respectively, and leukocytes were within the normal range. The renal computed tomography (CT) scan demonstrated renal asymmetry (the right kidney was larger than the left kidney), nephrocalcinosis and multiple kidney stones, one of which was causing right ureter hydronephrosis (Figure 1), leading to urosepsis. Blood culture analysis showed positive results for *Escherichia coli*, so the patient was started on ceftriaxone treatment, and a ureteral stenting was inserted. The patient’s condition improved, and she was discharged from the hospital with oral antibiotics.

The patient was followed up by the urology team, and the ureteral double-J stent was removed three months after the hospital discharge. The follow-up renal CT scan identified bilateral residual lithiasis, and a nephrology consultation was requested to study the cause of nephrocalcinosis.

**Figure 2:**
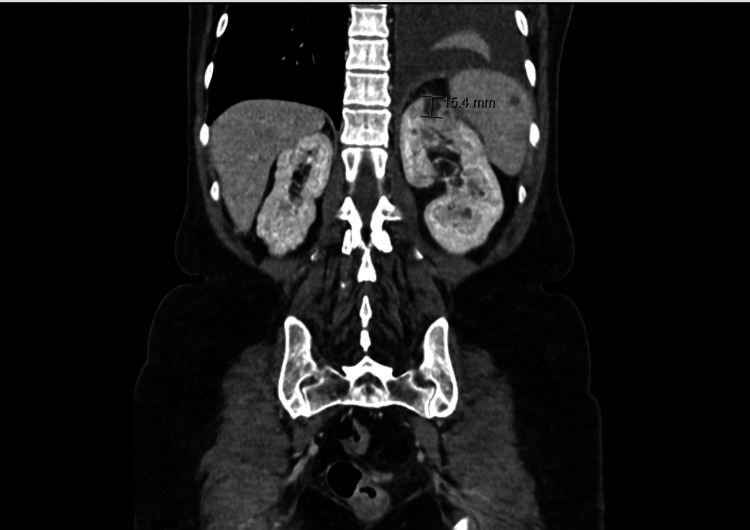
CT showing microabscesses in the left kidney (2022)

**Figure 3:**
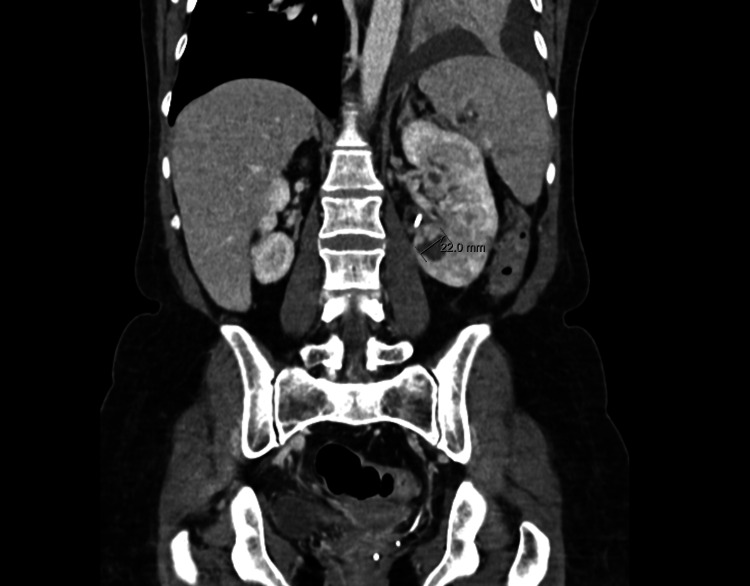
CT showing microabscesses in the left kidney (2022)

One year later, she was admitted to the hospital with left lumbar pain and fever and was diagnosed with acute obstructive pyelonephritis, caused by a 1 cm calculus in the proximal ureter. She spent three days in the ICU with antibiotic administration and underwent ureteral stenting. She was submitted to a reposition of a double-J stent 17 days later due to the development of renal microabscesses, as shown in Figures 2-3. The urine and blood cultures were *E. coli* positive. She was treated with antibiotics for 21 days, more specifically, one daily dose of ceftriaxone (2 g) for four days, followed by five days of piperacillin (4 g)/tazobactam (0.5 g) every eight hours and one dose of ceftriaxone (2 g) for 10 days. Infectious disease consultations guided the management of the medication.

The follow-up consultation with Nephrology led to the suspicion of PHPT, confirmed by the blood analysis that exhibited elevated serum calcium levels adjusted for albumin, high levels of PTH and normal 24-hour urine calcium (Table 1). The high levels of PTH and calcium could be caused by a deficit of vitamin D. However, the patient showed a vitamin D level of 24 ng/mL, which is lower than the standard values but not considered a deficit, so we proceeded to analyze the parathyroid.

**Table 1:** Laboratory values

Test	Result	Reference range
Hemoglobin	12.9	11.9-15.6 g/dL
White cell count	3.6	4.0-11.0x10^3^/uL
Platelet	234	150-400x10^3^/uL
C-reactive protein	1.6	<5.0 mg/L
Parathyroid hormone	213.06	18.5-88.0 pg/mL
Na	140	136-145 mmol/L
Potassium	4.7	3.5-5.1 mmol/L
Urea	44	19-49 mg/dL
Creatinine	0.9	0.6-1.20 mg/dL
Calcium	12.4	8.3-10.6 mg/dL
Phosphate	2.6	2.4-5.1 mg/dL
Magnesium	19	16-26 mg/L
Vitamin D	24	<10 ng/mL
24-hour urine calcium	268	100-300 mg/24 h

The thyroid ultrasound showed a 18x9x13 mm size parathyroid adenoma.

**Figure 4:**
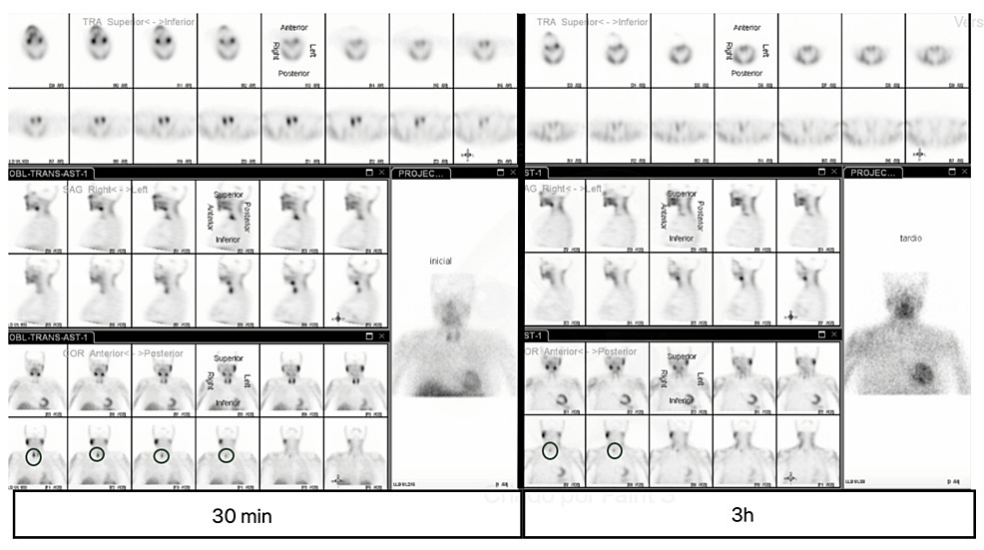
Parathyroid scintigraphy showing hyperfunctioning parathyroid tissue posterior to the inferior pole of the right thyroid lobe

**Figure 5:**
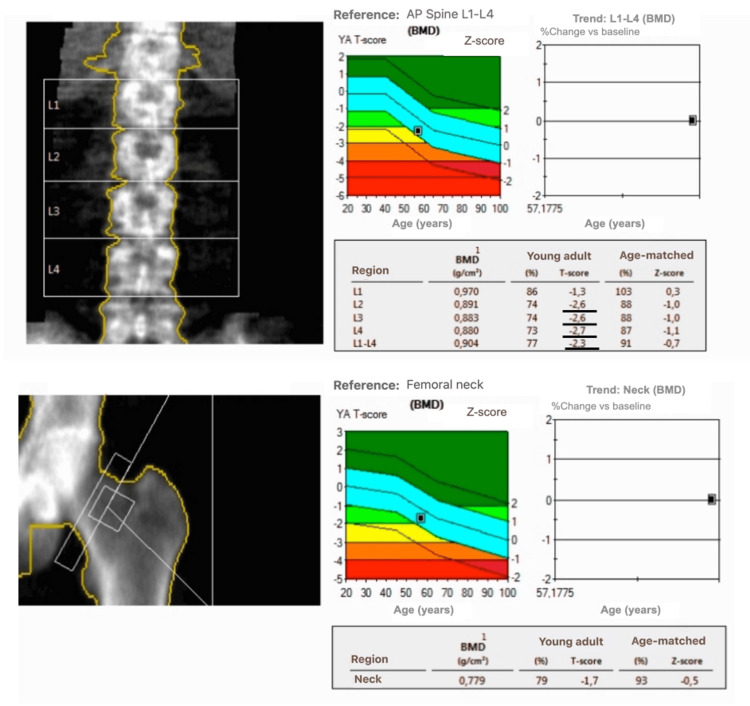
Bone densitometry showing signs of osteoporosis BMD, bone mineral density

In the pre-operative evaluation, a parathyroid scintigraphy was performed and that confirmed the presence of hyperfunctioning parathyroid tissue posterior to the inferior pole of the right thyroid lobe (Figure 4). The bone densitometry analysis demonstrated signs of osteoporosis (Figure 5).

The patient underwent a right lower parathyroidectomy in February 2023. The patient’s condition improved postoperatively, showing a decline in the blood serum calcium and PTH levels to the normal range values. Moreover, the patient has not experienced UTIs or acute renal colic since the surgery, demonstrating an overall recovery.

## Discussion

PHTP is usually diagnosed while the patients are still asymptomatic, but when the disorder manifests, the most prevalent symptoms are nephrolithiasis, nephrocalcinosis and fractures​​​​​ [1-5]. PHTP shows a significant preponderance in females and is diagnosed in patients above 50 years of age [1,2,4]. The diagnosis starts with clinical suspicion and through blood workup for calcium, PTH and vitamin D. The typical signs include high levels of calcium paired with high levels of PTH [1,6]​​​​​​. However, some patients do not present an increase in PTH levels (inappropriately normal levels), so the calcium rise is the stronger indicator.

Parathyroidectomy is considered the first line of therapy due to the minimal risk of recurrence and a relatively low complication rate [4,7]. Parathyroid adenomas are responsible for 85%-90% of all PHPT cases, multiglandular disease for 10%-15% of cases and parathyroid carcinomas cause 1% of the cases [1].

Parathyroidectomy is recommended if the patient exhibits blood calcium levels that are higher than 1 mg/dL above the superior limit range or the estimated glomerular rate is inferior to 70 mL/min [4,7]. It is also recommended if a patient has a T-score below -2.5 in any bone and/or pathologic fracture [4]. Recovery is successful if the calcium levels return to normal within six months after surgery [4,7]. Patients who cannot undergo surgery safely or who do not recover within six months can be started on medical therapy with oral calcimimetic cinacalcet [2,4]. This is considered a valid option for controlling serum calcium levels and lessening the patient's symptoms.

## Conclusions

PHPT is normally asymptomatic, but nephrocalcinosis can indicate an underlying cause and a thorough investigation must be performed. In this case, it was described as an atypical manifestation of severe symptoms of PHPT, with a delayed diagnosis that caused a disruption to the patient’s quality of life. The clinical analysis demonstrated high serum calcium and PTH levels, together with imaging of the kidneys showing irregular morphology and several kidney stones. The diagnosis was confirmed after the thyroid ultrasound exhibited the presence of an adenoma. The adenoma was removed by parathyroidectomy, and the patient’s condition improved, decreasing the levels of calcium and PTH to the baseline values.

The presented clinical study emphasizes the crucial role of timely investigation and diagnosis in preventing further complications. However, the health system cannot always meet the urgency of the cases for numerous reasons, having the need for alternative solutions. Family physicians could expedite the process by conducting the initial analysis of the pathology and subsequent follow-up examinations, for instance, recommending blood and urine analyses to assess calcium, PTH, and vitamin D levels. Based on the results, the decision to move forward with parathyroid imaging can be considered. This can reduce the waiting period between tests, facilitate early diagnosis, and improve patient outcomes.
